# Phase angle obtained via bioelectrical impedance analysis and objectively measured physical activity or exercise habits

**DOI:** 10.1038/s41598-022-21095-6

**Published:** 2022-10-14

**Authors:** Yosuke Yamada, Tsukasa Yoshida, Haruka Murakami, Ryoko Kawakami, Yuko Gando, Harumi Ohno, Kumpei Tanisawa, Kana Konishi, Tripette Julien, Emi Kondo, Takashi Nakagata, Hinako Nanri, Motohiko Miyachi

**Affiliations:** 1 Department of Physical Activity Research, National Institute of Health and Nutrition, National Institutes of Biomedical Innovation, Health and Nutrition, 1-23-1, Toyama, Shinjuku-Ku, Tokyo, 162-8636 Japan; 2 Faculty of Sport and Health Science, Ritsumeikan University, Kusatsu, Shiga 525-8577 Japan; 3 Faculty of Sport Sciences, Waseda University, Tokorozawa, Saitama 359-1192 Japan; 4 Faculty of Sport Science, Surugadai University, Hanno, Saitama 357-8555 Japan; 5 Depatment of Nutrition, Faculty of Health Care, Kiryu University, Midori, Gunma 379-2392 Japan; 6 Faculty of Food and Nutritional Sciences, Toyo University, Oura, Gunma 374-0193 Japan; 7 Center for Interdisciplinary AI and Data Science, Ochanomizu University, Bunkyo, Tokyo, 112-8610 Japan; 8 Faculty of Health and Sport Sciences, University of Tsukuba, Tsukuba, Japan; 9 Japan Society for the Promotion of Science, Tokyo, Japan

**Keywords:** Predictive markers, Physiology, Ageing

## Abstract

The phase angle (PhA), measured via bioelectrical impedance analysis, is considered an indicator of cellular health, where higher values reflect higher cellularity, cell membrane integrity, and better cell function. This study aimed to examine the relationship between PhA and exercise habits or objectively measured physical activity. We included 115 people aged 32–69 years. The body composition and PhA were measured using a bioelectrical impedance device. Physical activity and sedentary behavior (SB) were assessed using a triaxial accelerometer. Exercise habits were also obtained through structured interviews, and participants were categorized into the no exercise habit (No-Ex), resistance training exercise habit (RT), or aerobic training exercise habit (AT) groups. Objectively measured moderate-to-vigorous physical activity or step count significantly correlated with PhA, independent of age, sex, height, percent body fat, body cell mass, and leg muscle power. In contrast, SB was not significant determinants of PhA. People who exercised regularly (RT or AT) had significantly higher PhA values than did those in the No-Ex group. Furthermore, the PhA was not significantly different between the RT and AT groups. Regularly engaging in exercise with moderate-to-vigorous intensity may improve or maintain muscle cellular health and muscle quality.

## Introduction

Bioelectrical impedance analysis (BIA) measures the impedance of the human body^1^, which consists of resistance (R) and reactance (Xc). The phase angle (PhA) can be calculated as [arctangent (Xc/R) × 180°/π]^2^. Raw impedance variables have attracted substantial interest in clinical nutrition^3–9^. PhA is considered an indicator of cellular health, with higher values indicating higher cellularity, cell membrane integrity, and better cell function^10,11^. Previous studies have indicated that PhA decreases during aging^12,13^ and is correlated with nutritional status^11,14,15^ and disease status^9,14,16–19^, muscle power or strength^6,10,20,21^, aerobic capacity^22^ gait ability, and physical fitness^2^, prospective fall^23^ and mortality^24^.

The BIA theories have been established since the 1930s^25,26^, and BIA has been used to assess human body composition since the 1960s^27–31^. BIA requires ethnicity-specific, sex-specific, age-specific, or health condition-specific models or equations to estimate the human body composition. However, the PhA is obtained directly from the BIA raw impedance data with the above mentioned equation; thus, the results are not biased by the choice of equations. Because over 90% of the total impedance is obtained from appendicular segments (a leg and an arm) in the whole-body BIA method, in which the electrical path is from the ankle to the wrist, the whole-body PhA mainly reflects the characteristics of the appendicular lean soft tissue (ALST). Because most ALSTs are skeletal muscle cell tissues^32^, PhA is an indicator of skeletal muscle cell quality. The revised version of the European Working Group on Sarcopenia in Older People notes the importance of muscle quality assessed by PhA obtained by BIA, as well as that assessed by magnetic resonance imaging (MRI) or computed tomography^33^.

Regular physical activity and limited sedentary behavior (SB) are important factors for the prevention of non-communicable diseases, such as type-2 diabetes, cardiovascular diseases, and breast and colon cancers^34–39^. Regular physical activity and SB contribute to maintaining healthy weight, general well-being, cardiovascular and muscular health. Regular physical activity and SB can be evaluated objectively using accelerometry-based activity monitors or subjectively through interviews or questionnaires^40–43^. The above-mentioned cellular health may be related to regular physical activity or SB, but studies examining the association between PhA and physical activity or SB in healthy adults are limited^44^. To our knowledge, this study is the first to examine the association between PhA and objectively measured physical activity and SB in a healthy population.

We hypothesized that a higher PhA, an indicator of better cellular health, is associated with exercise habits or a longer moderate-to-vigorous intensity physical activity (MVPA) duration and a shorter duration of SB in daily life. Here, we examined the relationship between the PhA and exercise habits, or physical activity and SB, which were objectively assessed by accelerometry-based activity monitors in Japanese adults.

## Methods

### Participants

We included 115 people (30 men and 85 women; mean and SD of age were, 55.3 ± 8.0 years old, and range of age was 32–69 years old). Participants were recruited from a longitudinal study at the National Institute of Health and Nutrition, Tokyo^45^. The inclusion criteria were as follows: (1) anthropometric variables were measured. (2) Physical activity was measured using accelerometer-based activity monitors. (3) Their PhA was measured using bioelectrical impedance spectroscopy (BIS). We excluded the subjects with cardiovascular, respiratory, neurological, metabolic or orthopedic disorders. This studies were reviewed and approved by the Institutional Review Board of the National Institute of Biomedical Innovation, Health and Nutrition (No KENEI-102). The participants provided their written informed consent to participate in this study. All methods were performed in accordance with the relevant guidelines and regulations.

### Anthropometry, body composition, and phase angle

Height, weight, and waist circumference were measured, and body mass index (BMI) was calculated as weight in kilograms divided by height in meters squared (kg/m^2^). The body composition was estimated, and the PhA was measured using the BIS (SFB7, ImpediMed, Pinkenba, Australia) as follows^10^: Two injectable electrodes were placed on the dorsal surface of the right hand and foot, and detecting electrodes were placed on the dorsum of the right wrist and ankle (Red Dot, 3M Health Care, MN, USA). BIS was measured in the supine position, between 8 and 10 A.M., and before any physical fitness test. Fat-free mass (FFM), body cell mass (BCM), and percent body fat (%fat) were obtained using the BIS software (Bio-imp version 5.5.0.1, ImpediMed). Participants were divided into low and high PhA groups based on the sex-specific median of the PhA results (5.4 for women and 6.5 for men).

### Physical activity measurement

A previously validated triaxial accelerometer (Actimarker EW4800, Panasonic, Osaka, Japan) was used to track intensities of physical activity^46–49^. All participants were asked to wear a triaxial accelerometer for 20 days. We used the data for 14 days, during which the accelerometer was worn continuously on waking until going to bed. The accelerometer’s technical features and estimated equation were fully detailed^46,47^. The accelerometer samples the acceleration at a rate of 20 Hz, with a performance of zero to twice the momentum of gravity. It keeps track of the SD of the three-dimensional vector norm of the composite acceleration for a minute. In a study of healthy adults, the vector norm was significantly associated (R^2^ = 0.86) with oxygen uptake (VO_2_) during walking and running at seven different paces spanning from 40 to 160 m min^−1^ and during seven common activities: food preparation, self-care while upright, replacing clothing, cleaning dishes, eating supper, vacuuming, and doing laundry^46,50^. The metabolic equivalent of tasks (MET) and step counts were was obtained at one-minute intervals. MVPA was defined as MET ≥ 3.0. The light intensity physical activity (LPA) was defined as 1.5 to 2.9 METs, and SB was defined as any waking behavior characterized by ≤ 1.5 METs^51,52^.

### Exercise habits

Engaging in regular exercise habits was interviewed by well-trained staff using the structured method of the National Nutrition Survey in Japan^53–55^. The participants were asked whether they currently exercised (over 30 min per session, twice a week for 3 months). Participants who answered “yes” were classified as having exercise habits (exercised regularly). Participants who answered “no” were classified as participants without exercise habits (non-Ex No-Ex group). Moreover, we divided the participants who have exercise habits into engaging resistance training (RT group) or only aerobic exercises (AT group).

### Blood samples

Blood samples were collected from participants following an overnight fast for at least 10 h between 8 and 10 A.M.^55^. Venous blood withdrawn from the antecubital vein was collected into tubes without additives or EDTA and immediately centrifuged at 3000 rpm for 20 min to obtain serum or plasma. The levels of glucose and glycated hemoglobin (HbA1c) in plasma, total cholesterol, high-density lipoprotein (HDL) cholesterol, and triglycerides in the serum were determined^55^.

### Grip strength

Grip strength tests were conducted after BIA measurements. Maximal GS was measured using a Smedley Hand Dynamometer (Grip-D, TKK5401; Takei Scientific Instruments, Niigata, Japan), as described elsewhere^56^. When performing the measurement, subjects were instructed to maintain a standard bipedal position for the duration of the test. The involved arm was placed in complete extension with the dynamometer not touching any other part of the body, except the hand being measured. The width of the handle was adjusted to ensure that, when the subject held the dynamometer, the second phalanx was against the inner stirrup. Two trials, separated by a brief rest, were allowed for each hand alternately, and the highest value was recorded as the result. Subjects were encouraged to exert themselves maximally during each effort.

### Statistical analyses

The results are presented as the mean ± SDs. One-way analysis of variance (ANOVA) and analysis of covariance (ANCOVA) was used to compare physical characteristics and physical activities between the low and high PhA groups. Age and sex were set as covariates for the ANCOVA. Pearson’s correlation coefficients were calculated, and partial correlation coefficients were calculated between the PhA and other variables, with age and sex as control variables. Correlations presenting coefficients between, 0.00–0.29, 0.30–0.49, 0.50–0.69, 0.70–0.89, and 0.90–1.0, were considered “negligible”, “low”, “moderate”, “high” and “very high”, respectively^57^. Multiple linear regression analyses were conducted with PhA as the objective variable and age, sex, height, %fat, BCM, leg muscle power, MVPA, step counts, or regular exercise habits as the explanatory variables while avoiding multicollinearity (< 5 variance inflation factor [VIF]). An alpha of 0.05 was used for statistical significance for all analyses. All analyses were performed using SPSS 22.0 (IBM Inc., USA).

## Results

The physical characteristics and activities of the participants are presented in Table 1. The ANOVA results showed that the high PhA group was younger and had higher BMI, BCM, calf circumference, leg muscle power, MVPA, and step counts than the low PhA group (P < 0.05). In addition, men had higher PhA values than women (6.5° ± 0.7° vs. 5.4 ± 0.5°, P < 0.001). The ANCOVA results show that the high PhA group had higher values of weight, BMI, FFM, BCM, calf circumference, handgrip strength, leg muscle power, MVPA, and step counts than the low PhA group after adjustment for age and sex (P < 0.05). Higher BMI in the higher PA group was due to higher body cell mass (P < 0.01), but not due to percent body fat difference (P > 0.4).

**Table 1:** Physical characteristics and physical activities of the participants (mean ± SD).

	Low PhA group	High PhA group	ANOVA	ANCOVA
	(n = 57)	(n = 58)	p-value	p-value
Age (y)	57.7 ± 7.9	53.1 ± 7.5	**0.002**	**–**
PhA (deg)	5.3 ± 0.6	6.1 ± 0.7	** < 0.001**	** < 0.001**
Height (cm)	161.2 ± 8.5	161.2 ± 7.5	0.988	0.438
Weight (kg)	57.0 ± 9.8	60.4 ± 9.7	0.067	**0.024**
BMI (kg m^−2^)	21.8 ± 2.4	23.1 ± 2.9	**0.007**	**0.002**
Waist circumference (cm)	79.1 ± 7.8	80.8 ± 8.8	0.276	0.060
Percent body fat (%)	23.7 ± 6.0	22.8 ± 6.4	0.468	0.538
FFM (kg)	43.6 ± 9.0	46.6 ± 8.9	0.075	**0.007**
BCM (kg)	25.0 ± 5.0	27.5 ± 5.2	**0.009**	** < 0.001**
SMI (kg m^−2^)	6.13 ± 1.69	6.52 ± 1.65	0.212	**0.031**
Calf circumference (cm)	34.5 ± 2.4	35.6 ± 2.4	**0.013**	**0.016**
Hand grip strength (kg)	28.9 ± 8.3	30.8 ± 6.3	0.169	**0.032**
Leg muscle power (W kg^−1^)	17.1 ± 5.0	19.3 ± 4.9	**0.018**	**0.035**
Triacylglycerol (mg dL^−1^)	79.7 ± 33.8	85.8 ± 43.4	0.400	0.146
HDL (mg dL^−1^)	69.1 ± 15.3	67.8 ± 23.0	0.723	0.719
Fasting glucose (mg dL^−1^)	88.0 ± 20.0	86.3 ± 7.8	0.539	0.922
HbA1c (%)	5.5 ± 0.5	5.4 ± 0.3	0.307	0.935
HOMA-IR	0.78 ± 0.58	0.88 ± 0.46	0.329	0.219
SBP (mmHg)	117.2 ± 15.6	118.6 ± 12.1	0.591	0.197
DBP (mmHg)	72.4 ± 10.8	72.1 ± 8.7	0.881	0.792
Sleeping time (min)	387 ± 66	381 ± 76	0.651	0.367
SB (min)	656 ± 114	646 ± 117	0.636	0.606
LPA (min)	336 ± 103	339 ± 95	0.865	0.474
MVPA (min)	61.3 ± 25.3	74.4 ± 33.2	**0.020**	**0.045**
Step counts (day^−1^)	9386 ± 2975	10,806 ± 3238	**0.016**	**0.037**

Age and sex were the controlling variables of ANCOVA.

PhA, phase angle; BMI, body mass index; FFM, fat-free mass; SMI, skeletal muscle mass index; HDL, High-density lipoprotein; SBP, systolic blood pressure; DBP, diastolic blood pressure; SB, sedentary behavior (< 1.5 METs); LPA, light intensity physical activity (1.5–2.9 METs); MVPA, moderate-to-vigorous physical activity (≥ 3.0 METs).

Significant values are given in bold.

**Figure 1:**
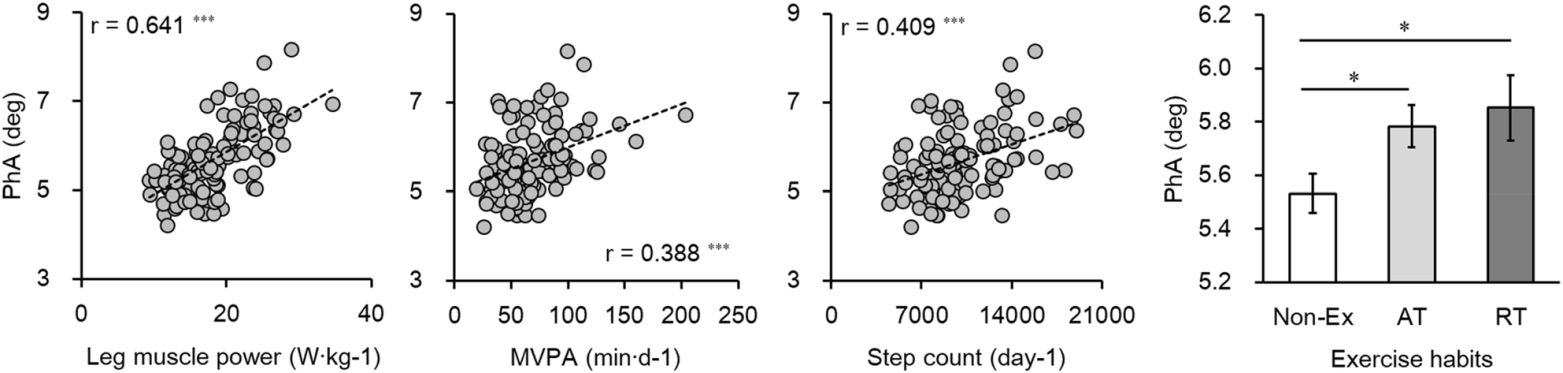
Relationship between leg muscle power, moderate-to-vigorous physical activity (MVPA), step count, or exercise habits with phase angle (PhA). *Significant difference between group P < 0.05.

Table 2 shows the Pearson and partial correlation coefficients between PhA and other variables. The PhA was negatively and significantly correlated with age (r = − 0.361, P < 0.001). BMI, percent body fat, FFM, BCM, leg muscle power, MVPA, and step counts were significantly correlated with PhA in both Pearson and partial correlation analyses (P < 0.05). The relationship between PhA and leg muscle power, MVPA, and step count is shown in Fig. 1. In addition, we compared the PhA values between the No-Ex, AT, and RT groups using ANCOVA and found that the AT and RT groups had significantly higher PhA (P < 0.05) than the No-Ex group, even after adjustment for age and sex (Fig. 1). In contrast, there was no significant difference between the AT and RT groups (P > 0.05).

**Table 2:** Correlation coefficients between PhA and other variables.

	Pearson corr	Partial corr
Age (y)	− 0.361***	
Height (cm)	0.410***	− 0.177
Weight (kg)	0.495***	0.087
BMI (kg m^−2^)	0.331***	0.211*
Waist circumference (cm)	0.194	0.071
Percent body fat (%)	− 0.555***	− 0.234*
FFM (kg)	0.656***	0.226*
BCM (kg)	0.715***	0.378***
SMI (kg m^−2^)	0.678***	0.174
Calf circumference (cm)	0.424***	0.127
Hand grip strength (kg)	0.651***	0.169
Leg muscle power (W kg^−1^)	0.641***	0.259**
Triacylglycerol (mg dL^−1^)	0.209*	0.07
HDL (mg dL^−1^)	− 0.286**	− 0.032
Fasting glucose (mg dL^−1^)	0.106	− 0.029
HbA1c (%)	− 0.071	− 0.009
HOMA-IR	0.134	0.035
SBP (mmHg)	0.162	0.054
DBP (mmHg)	0.283**	0.031
Sleeping time (min)	− 0.001	− 0.076
SB (min)	0.115	− 0.091
LPA (min)	− 0.250**	0.094
MVPA (min)	0.388***	0.248**
Step counts (day^−1^)	0.409***	0.265**

Age and sex were the controlling variables of partial correlation.

PhA, phase angle; BMI, body mass index; FFM, fat-free mass; SMI, skeletal muscle mass index; HDL, High-density lipoprotein; SBP, systolic blood pressure; DBP, diastolic blood pressure; SB, sedentary behavior (< 1.5 METs); LPA, light intensity physical activity (1.5–2.9 METs); MVPA, moderate-to-vigorous physical activity (≥ 3.0 METs); * P < 0.05; ** P < 0.01; *** P < 0.001.

The results of multiple regression analyses are shown in Table 3. All variables in both models have < 5. BCM, leg muscle power, MVPA, step counts, and regular exercise habits were selected as significant predictors of the between-individual difference in PhA, independent of age, sex, height, and percent body fat (P < 0.05).

**Table 3:** Multiple linear regression analyses for phase angle (deg).

Factors included	Unstandardized	Standardized	p-value
	B (95% CI)	β	
**Model 1 (adjusted R**^**2**^** = 0.695)**			
Constant	9.441 (6.842, 12.041)		< 0.001
Age (y)	− 0.010 (− 0.021, 0.002)	− 0.100	0.103
Sex (men = 1, women = 0)	0.296 (− 0.04, 0.632)	0.172	0.083
Height (cm)	− 0.042 (− 0.058, − 0.027)	− 0.446	< 0.001
Percent body fat (%)	− 0.004 (− 0.022, 0.014)	− 0.030	0.681
BCM (kg)	0.098 (0.071, 0.126)	0.680	< 0.001
Leg muscle power (W kg^−1^)	0.043 (0.018, 0.068)	0.290	0.001
MVPA (min)	0.004 (0.001, 0.006)	0.145	0.012
**Model 2 (adjusted R**^**2**^** = 0.695)**			
Constant	8.848 (7.065, 13.10)		< 0.001
Age (y)	− 0.009 (− 0.031, − 0.007)	− 0.092	0.140
Sex (men = 1, women = 0)	0.25 (0.151, 0.908)	0.146	0.149
Height (cm)	− 0.039 (− 0.051, − 0.014)	− 0.414	< 0.001
Percent body fat (%)	− 0.005 (− 0.039, − 0.003)	− 0.039	0.593
BCM (kg)	0.097 (0.022, 0.061)	0.673	< 0.001
Leg muscle power (W kg^−1^)	0.044 (0.022, 0.061)	0.297	0.001
Step counts (day^−1^)	0.000035 (0.000008, 0.000062)	0.147	0.012
**Model 3 (adjusted R**^**2**^** = 0.688)**			
Constant	10.254 (7.68, 12.83)		< 0.001
Age (y)	− 0.014 (− 0.025, − 0.002)	− 0.144	0.022
Sex (men = 1, women = 0)	0.4 (0.056, 0.745)	0.233	0.023
Height (cm)	− 0.044 (− 0.06, − 0.028)	− 0.463	< 0.001
Percent body fat (%)	− 0.006 (− 0.024, 0.012)	− 0.05	0.498
BCM (kg)	0.096 (0.068, 0.123)	0.661	< 0.001
Leg muscle power (W kg^−1^)	0.04 (0.014, 0.065)	0.265	0.003
Exercise habits (yes = 1, no = 0)	0.169 (0.005, 0.333)	0.111	0.044

BCM, body cell mass; CI, confidence interval.

## Discussion

In the present study, daily MVPA, step count, and exercise habits, FFM, BCM, calf circumference, handgrip strength, and leg muscle power are associated with PhA. The objectively measured MVPA or step count was significantly associated with PhA in healthy adults, independent of age, sex, height, percent body fat, BCM, and leg muscle power. Conversely, SB and LPA were not significant determinants of PhA. Moreover, people who exercised had significantly higher PhA values than those in the No-Ex group. Furthermore, the PhA value was not significantly different between the RT and AT groups in our study.

In the present study, age was negatively associated with PhA, and men (mean ± SD; 6.5 ± 0.7) had a higher PhA than women (5.4 ± 0.5) (Table 3). The results was consistent with previous studies^5,13^. Moreover, the leg muscle power was positively associated with PhA even after controlling for age and sex. These observations were consistent with previous studies^5,13^. PhA was also positively associated with BCM and FFM and negatively associated with body fat percentage in the current population. The range of PhA was 4.2 to 8.2 which was also consistent with previous studies^2,5^.

Regular physical activity was assessed via objective and subjective methods. For objective assessment, a previously validated triaxial accelerometer was used to assess daily step counts, MVPA, LPA, and SB^46–49^. We found that step counts and MVPA was significantly and positively correlated with PhA, even after adjusting for age, sex, height, percent body fat, BCM, and leg muscle power. In contrast, SB was not significantly associated with PhA. As a subjective method, the exercise habits were assessed by the interview based on the structured method of the National Nutrition Survey in Japan^53–55^. In addition to step count or MVPA, subjectively assessed exercise habits were also significantly associated with PhA (Table 3, Model 3). Furthermore, the exercise modality (RT or AT) did not affect the results of the PhA (Fig. 1).

Mundstock reviewed previous studies examining the association between PhA and physical activity^44^. In the healthy adult population review, most of the previous studies applied a randomized controlled trial design with RT mode and subjective physical activity assessment. Ribeiro et al. found that 8-week RT significantly improved the PhA^58^, and Souza et al. also found that 12-week RT improved PhA^59^. Most recently, Otsuka et al. examined the effect of 24-wk moderate-intensity RT on thigh PhA in middle-aged and older adults and found that moderate-intensity RT also improved PhA (+ 0.3°)^60^. In addition, the change in PhA significantly correlated with the change in thigh muscle cross-sectional area (CSA) measured by MRI. Otsuka et al.^60^ also found that PhA is correlated with the ratio between intermuscular adipose tissue and thigh muscle CSA, suggesting that PhA reflects muscle quality. Thus, RT, which induces muscle hypertrophy, improves the PhA in the segments. The present study also showed that people who engaged in regular RT had significantly higher PhA than those who did not have any exercise habits.

Several researches have assessed the effect of daily physical activity on PhA^61,62^. In addition, Cupisti et al. examined 50 hemodialysis patients and found that the daily energy expenditure estimated using the SenseWear Armband was positively related to PhA (r = 0.40, P < 0.01)^63^. In the healthy adult population, we found that MVPA and daily step counts were positively associated with PhA (r = 0.388 and r = 0.409, respectively; P < 0.001). The current results are consistent with those findings.

PhA was not significantly different between the RT and AT groups in participants who exercised regularly in the present study. No other study has compared the effects of RT and AT on the PhA; therefore, further studies are required. In addition, the present study has some limitations. This is a cross-sectional study; thus, cause-and-effect logic is not clear. Hence, longitudinal and intervention studies are also required. Furthermore, the current sample size is small and further large cohort study are required. Nevertheless, our results suggest that regularly engaging in exercises with moderate-to-vigorous intensities may improve or maintain muscle cellular health. However, PhA was not significantly correlated with SB or LPA, which may indicate that preventing SB by engaging in LPA is not sufficient to improve muscle cellular health.

## Data Availability

The datasets used and/or analyzed during the current study available from the corresponding author on reasonable request.
